# Metacognitive beliefs of efficacy about daily life situations and use of cognitive strategies in amnestic mild cognitive impairment: a cross-sectional study

**DOI:** 10.3389/fpsyg.2024.1275678

**Published:** 2024-02-13

**Authors:** Grigoria Bampa, Despina Moraitou, Panagiota Metallidou, Elvira Masoura, Georgia Papantoniou, Maria Sofologi, Georgios Kougioumtzis, Efthymios Papatzikis, Magdalini Tsolaki

**Affiliations:** ^1^Laboratory of Psychology, Department of Cognition, Brain and Behavior, School of Psychology, Aristotle University of Thessaloniki, Thessaloniki, Greece; ^2^Laboratory of Neurodegenerative Diseases, Center of Interdisciplinary Research and Innovation (CIRI–AUTH), Balcan Center, Thessaloniki, Greece; ^3^Laboratory of Psychology, Department of Early Childhood Education, School of Education, University of Ioannina, Ioannina, Greece; ^4^Institute of Humanities and Social Sciences, University Research Centre of Ioannina (URCI), Ioannina, Greece; ^5^Department of Turkish and Modern Asian Studies, National and Kapodistrian University of Athens, Athens, Greece; ^6^Department of Psychology, Neapolis University Pafos, Pafos, Cyprus; ^7^Department of Early Childhood Education and Care, Oslo Metropolitan University, Oslo, Norway; ^8^Bright Start Foundation for Maternal and Child Health, Geneva, Switzerland; ^9^School of Medicine and Health, Khalifa University, Abu Dhabi, United Arab Emirates; ^10^School of Medicine, Aristotle University of Thessaloniki, Thessaloniki, Greece; ^11^Greek Association of Alzheimer’s Disease and Related Disorders (GAADRD), Thessaloniki, Greece

**Keywords:** metacognition, self-efficacy, mild cognitive impairment, cognitive strategies, attention

## Abstract

Metacognition, the ability to monitor and regulate cognitive processes, is essential for individuals with Mild Cognitive Impairment (MCI) to accurately identify their deficits and effectively manage them. However, previous studies primarily focused on memory awareness in MCI, neglecting other domains affected in daily life. This study aimed to investigate how individuals with MCI perceive their abilities to handle various cognitively challenging situations representing real-life scenarios and their use of compensatory strategies. Thus 100 participants were recruited, including 50 with amnestic MCI with multiple deficits (aMCI) and 50 cognitively healthy controls (HC) matched in age and education. Participants completed three metacognitive scales assessing self-perceived efficacy in everyday life scenarios and one scale evaluating use of cognitive strategies. Results indicated that aMCI participants reported significantly lower self-efficacy in memory and divided-shifted attention scenarios compared to HC. Surprisingly, no significant group differences were found in the self-reports about the use of cognitive strategies. This suggests a potential gap in understanding or applying effective strategies for compensating cognitive deficits. These findings emphasize the importance of cognitive training programs targeting metacognitive knowledge enhancement and practical use of cognitive strategies that could enhance the quality of life for individuals with MCI.

## 1 Introduction

Metacognition encompasses the capacity to oversee and control our cognitive functions. Metacognitive knowledge, one of the main components of metacognition, encompasses an individual’s awareness and beliefs about cognitive processes, including their own (Flavell, 1979). Metacognitive knowledge and beliefs play a critical role in monitoring and regulating cognitive abilities (Schraw and Moshman, 1995; Nelson, 1996) something which becomes particularly important in the context of mild cognitive impairment (MCI).

MCI is considered a stage between normal cognitive aging and dementia, characterized by a noticeable decline in cognitive functions but not severe enough to interfere with independence in daily life (Petersen, 2004). MCI is classified into amnestic (aMCI) and non-amnestic (naMCI) subtypes, based on whether the primary cognitive deficit involves memory or other cognitive domains, respectively (Petersen et al., 2014). An additional subdivision is whether individuals present deficits in a single cognitive domain or across multiple cognitive domains. Thus, people with MCI may have difficulties in memory, attention, and/or other cognitive domains, which could be associated with their metacognitive abilities (Perrotin et al., 2007; Isingrini et al., 2008). As a result, they may struggle to accurately evaluate their cognitive performance or employ effective strategies.

Impaired awareness of one’s cognitive skills can influence the regulation of cognitive behavior leading to inefficient use of compensatory strategies and unsuccessful attempts to manage age-related cognitive deficits (Irak and Çapan, 2018), ultimately affecting their cognitive performance and overall quality of life. In contrast, intact cognitive awareness enhances the comprehension of factors influencing success or failure (McGillivray and Castel, 2011; Siegel and Castel, 2019), and it is crucial for detecting strengths and/or weaknesses to adjust cognitive resources and improve performance (Hertzog and Dunlosky, 2011). Furthermore, research has shown that understanding older adults’ subjective beliefs about their cognitive abilities has important implications for how they handle cognitive challenges, such as their willingness to engage in cognitively demanding situations, the effort they put forth, the strategies they use, and ultimately their performance (Castel et al., 2009; Beaudoin, 2018; Cherry et al., 2019). The link between metacognitive awareness−recognition and understanding of one’s cognitive strengths and weaknesses−and these subjective beliefs about cognitive abilities is intricate. Metacognitive awareness helps individuals accurately gauge their competence and informs their self-efficacy beliefs, influencing their approach to cognitive tasks (Hertzog and Dunlosky, 2011; McGillivray and Castel, 2011; Irak and Çapan, 2018; Siegel and Castel, 2019). Moreover, Bandura’s concept of self-efficacy—our belief in our ability to accomplish cognitive tasks—has significant overlaps with metacognitive awareness and beliefs about cognitive abilities (Bandura, 1977). This perception greatly influences motivation and persistence in task execution (Cera et al., 2013). A low self-efficacy may result in avoidance behaviors, amplified stress, depressive symptoms, and the reinforcement of a negative self-efficacy loop (Bandura, 1986; Carretti et al., 2011; O’Shea et al., 2016). In other words, if an individual perceives their ability to handle certain cognitively demanding situations as low (a low self-efficacy belief), they may evade such situations, consequently reinforcing their beliefs of cognitive incompetence. However, it is crucial to emphasize that while higher self-efficacy beliefs can lead to greater engagement in cognitive challenges, a willingness to apply cognitive resources effectively, and an overall more adaptive approach to cognitive tasks, these beliefs must be in line with accurate metacognitive awareness—i.e., aligned with one’s actual cognitive skills. An overestimation of cognitive abilities due to impaired metacognitive awareness can lead to negative consequences, such as risky behaviors, and suboptimal cognitive strategies, further underscoring the intricate interplay between metacognitive awareness and self-efficacy.

The importance of metacognition in preserving cognitive functioning and effectively employing coping mechanisms has led researchers to examine metacognitive awareness in MCI. Research so far has shown mixed results (Seelye et al., 2010). This variance could potentially be attributed to the diversity within the MCI diagnosis itself (such as aMCI versus non-aMCI), or the absence of a universally accepted “gold standard” for assessing metacognitive awareness. Typically, the studies that have utilized offline measures to assess metacognitive awareness, such as self-reports and patient-informant discrepancy studies have shown that individuals with MCI have impaired insight into their cognitive abilities (Vogel et al., 2004; Galeone et al., 2011; Clare et al., 2013). Additionally, the degree of metacognitive awareness impairment has been associated with a heightened risk of progression to Alzheimer’s disease dementia (ADD) (Bastin et al., 2021). Therefore, incorporating the assessment of metacognitive awareness in individuals with MCI into neuropsychological screening processes could be beneficial. This approach would establish a foundation for more effective interventions, aimed at improving their quality of life and potentially slowing down progression toward dementia.

Regarding the use of cognitive strategies, past studies indicate that older adults naturally employ a range of compensatory strategies to cope with cognitive decline; however, it is observed to rise with age and before an MCI diagnosis, yet it is less prevalent among individuals diagnosed with dementia (Anderson and Schmitter-Edgecombe, 2010; Bottiroli et al., 2017; Farias et al., 2018). Farias et al. (2018) found in their study that participants with MCI exhibited no differences in their use of compensatory strategies compared to healthy older adults. Additionally, a more recent study by Lin et al. (2020) compared the usage of compensatory strategies among participants with different MCI subtypes (amnestic and non-amnestic) and found no differences between the two groups. These findings indicate that individuals with MCI may have difficulties using strategies effectively to compensate for their cognitive challenges. Possible explanations could include a lack of awareness of the effectiveness of these strategies, uncertainties about their proper application, or an overall lack of awareness of their cognitive deficits. All these possibilities point to potential deficits in metacognitive monitoring and/or control processes, underlining the necessity for further research in this area.

In summary, metacognitive awareness significantly influences the management of MCI. An accurate assessment of this awareness could lead to more effective interventions, potentially decelerating the progression toward dementia and improving overall quality of life.

### 1.1 Aim and hypothesis

Several studies investigated metacognitive awareness in people with MCI. However, most of them have exclusively focused on metamemory or adopted a deficit-oriented approach, which primarily considers the presence or frequency of cognitive pitfalls. Others have based their assessments on discrepancies between the individual’s and informant’s perceptions. These methods, although valuable in enhancing our understanding, fall short in providing insights into how these individuals perceive their abilities. Therefore, the aim of the present study is to further explore how individuals with MCI perceive their abilities to manage a variety of cognitively challenging situations that reflect everyday life scenarios, along with their use of compensatory strategies. To achieve this, the study will assess self-efficacy beliefs related to everyday cognitive abilities to evaluate metacognitive knowledge across various domains. It is hypothesized that individuals with MCI will report lower average efficacy ratings regarding their performance in everyday life scenarios compared to their healthy counterparts, demonstrating an awareness of their cognitive struggles (Hypothesis 1). As for the frequency of strategy usage, drawing from a previous study (Farias et al., 2018) we expected no differences between the two groups (Hypothesis 2). While it might initially appear contradictory that individuals, despite awareness of their cognitive difficulties, do employ more strategies, several factors may hinder this adaptation. These could include unawareness of the efficacy of cognitive strategies or a perception that the effort to learn and apply these strategies exceeds the perceived benefits, especially if their cognitive difficulties don’t drastically impede daily life. Therefore, this intriguing aspect warrants deeper exploration.

## 2 Materials and methods

### 2.1 Participants

Based on a power analysis that was conducted, using G*Power (Faul et al., 2007), a total sample size of at least 90 participants was recommended to achieve a power of.80. The study included 100 participants, 50 individuals diagnosed with aMCI and 50 healthy controls, 33 men and 67 women, with mean age of 61,98 (SD = 6,27) years and mean education of 14,95 (SD = 2,94) years. Participants needed to be native Greek speakers, over 50, with at least six years of education (see Table 1 for information about each group). They underwent a comprehensive neuropsychological assessment to distinguish healthy individuals from those with aMCI, based on Petersen’s criteria (Petersen et al., 2014) and DSM-V (American Psychiatric Association, 2013). However, the specific cognitive scores from this assessment are not included in this paper as they fall outside the scope of our current study. It is important to note that this study is part of a broader project wherein participants underwent supplementary neuropsychological tests. The differences in cognitive performance between the two groups based on these scores are detailed in another publication by our team (Bampa et al., 2023). The neuropsychological assessment included a variety of tools: The Geriatric Depression Scale (Yesavage et al., 1982; Fountoulakis et al., 1999), the Beck Depression Inventory (Beck et al., 1961), the Beck Anxiety Inventory (Beck et al., 1988), and the Short Anxiety Screening (Sinoff et al., 1999; Grammatikopoulos et al., 2010) were implemented to rule out affective disorders. The Neuropsychiatric Inventory (Cummings et al., 1994; Politis et al., 2004) was used to eliminate neuropsychiatric symptoms. The Mini Mental State Examination (Folstein et al., 1975; Fountoulakis et al., 2000) and the Montreal Cognitive Assessment (Nasreddine et al., 2005; Poptsi et al., 2019) were employed to examine the overall cognitive status, along with the Functional Cognitive Assessment (Kounti et al., 2006) to evaluate executive functions across six daily activities. Additionally, other standardized cognitive tests were utilized to evaluate memory, attention, executive functions, and language skills. Participants’ degree of cognitive decline was determined using the Global Deterioration Scale (GDS) (Reisberg et al., 1982). Based on this, those exhibiting no cognitive decline and normal functioning were classified as stage 1, while those with MCI were designated as stage 3. A comprehensive list of all employed neuropsychological tests can be found in the study by Tsolaki et al. (2017). The study specifically focused on participants displaying the amnestic subtype of MCI with multiple deficits, characterized by significant memory deficits along with minor impairments in other cognitive domains such as attention, language, or executive functions (Petersen, 2004). Given the accelerating global incidence of AD dementia and the fact that aMCI often precedes AD, the risk of future AD dementia is considerably higher in multi-domain aMCI (Petersen et al., 2014), hence the specific focus on this subtype.

**TABLE 1:** Participants’ demographic characteristics.

	aMCI (n = 50)	HC (n = 50)		
	**Mean (SD)**	**Mean (SD)**	**F**	**p**
**Demographics**				
Age	62.76(6.67)	61.20(5.78)	1.559	n.s.
Education	14.58(2.87)	15.32(2.99)	1.599	n.s.
Gender (f/m)	34/16	33/17	x^2^	n.s.

Exclusion criteria for both groups included: psychiatric disorders, substance abuse, brain injuries, neurological issues, diabetes, cardiovascular diseases’ factors, sensorimotor deficits, vitamin B12 deficiency, and cognitive complaints for the HC group.

Univariate analysis of variance (ANOVA) was used to analyze differences in age and education between groups, revealing no significant differences for age, F (1, 98) = 1.559, *p* = 0.215, or education, F (1, 98) = 1.559, *p* = 0.209. Chi-square analysis showed no significant differences in gender distribution, χ^2^(1) = 0.045, *p* = 0.832 (see Table 1).

### 2.2 Procedure

The study involved participants from the “Agia Eleni” day care center and Aristotle University of Thessaloniki, recruited with help from psychology interns. Each participant underwent a neuropsychological evaluation by experienced psychologists from the Greek Association of Alzheimer’s Disease and Related Disorders to determine cognitively healthy older adults and individuals with aMCI. Participants who voluntarily consented to join the study were individually scheduled for online appointments, necessitated by COVID-19 restrictions. The administration involved sharing a screen through platforms like Skype, Zoom, or Messenger, where the study’s questionnaires were presented and lasted approximately 15 to 20 min depending on the participant’s pace. The participant was requested to be in a quiet room without any distractions. The psychologist guided the participants, encouraging them to read and respond to each item. Clarity of questions was ensured, and the psychologist was available for any necessary explanations.

### 2.3 Ethics

The current study’s protocol was reviewed and approved by the Scientific Committee of ‘Alzheimer Hellas’ (Approval Code: 29/15-02-2017) and all ethical guidelines were followed in accordance with the 1964 Helsinki Declaration. In adherence to the European Union legislation effective from May 28, 2018, that allows the use of sensitive personal data for research objectives, demographic details such as age, gender, and level of education were gathered from the participants. Informed consent was obtained from all participants after providing them with information about the study’s aim and procedures.

### 2.4 Measures

#### 2.4.1 Metacognitive Knowledge for Everyday Memory (MKEM)

MKEM (Bampa et al., 2022) is a 12-item self-report measure designed to assess older adults’ metacognitive beliefs about everyday life scenarios related to memory function. For each scenario, participants were requested to estimate their degree of efficacy on a 4-point Likert scale, ranging from 1 “not at all” to 4 “very well” (example: Imagine that you want to tell a story that you read earlier in a book or in a newspaper. How well do you manage to remember details of that story, such as names, place, and time?). The scale has a one-factor structure and high internal reliability (α = 0.88), for a detailed overview see Supplementary Table 1.

#### 2.4.2 Metacognitive Knowledge for Everyday Attention (MKEA)

MKEA (Bampa et al., 2022) is a 12-item self-report measure designed to assess older adults’ metacognitive beliefs about everyday life scenarios related to attention. For each scenario, participants were requested to estimate their degree of efficacy on a 4-point Likert scale, ranging from 1 “not at all” to 4 “very well” (example: Imagine that you are at the bank, and you are waiting for your number to appear on the announcement table. How well do you manage to stay focused so that you do not lose your turn when your number appears?). The scale has a two-factor structure, with factors reflecting “Divided and Shifted Attention” (α = 0.74) and “Concentration” (α = 0.75), for a detailed overview see Supplementary Table 2.

#### 2.4.3 Metacognitive Knowledge for Everyday Executive Functions (MKEEFs)

Metacognitive Knowledge for Everyday Executive Functions (Bampa et al., 2022) is a 10-item self-report measure designed to assess older adults’ metacognitive beliefs about everyday life scenarios related to executive functions. For each scenario, participants were requested to estimate their degree of efficacy on a 4-point Likert scale, ranging from 1 “not at all” to 4 “very well” [example: Imagine that you have planned to go on a walk with a friend, but it starts raining. How well do you manage to think of an alternative plan considering the weather (i.e., sit in a cafeteria)?]. The scale has a two-factor structure, with factors reflecting “Planning” (α = 0.70) and “Inhibition” (α = 0.65), for a detailed overview see Supplementary Table 3.

#### 2.4.4 Multifactorial Memory Questionnaire – strategies subscale (MMQ)

Multifactorial Memory Questionnaire (Troyer and Rich, 2002) is a 57-item self-report scale developed to assess metamemory in older adults. It consists of three subscales: (1) Contentment (i.e., how one feels about one’s memory), (2) Ability (i.e., how one appraises one’s memory skills), and (3) Strategies (i.e., how frequently one uses cognitive strategies) and it has good psychometric properties. The scale was translated and partially adapted for the Greek population by Evdokia Emmanouilidou, Nikoleta Fratzi, and Despina Moraitou.^1^ For the purposes of the present study, we only used the Strategies subscale, which is a 19-item self-report questionnaire. The responses were given on a 5-Likert scale ranging from 0 (never) to 4 (always) with higher scores indicating more frequent use of strategies (example: How often do you make a list, such as a grocery list or a list of things to do?).

The structural validity and internal reliability of the MMQ-Strategies subscale were assessed in the present study based on the scores from the current sample. Exploratory factor analysis (EFA) employing Varimax rotation and Kaiser normalization was carried out. The number of extracted factors was determined based on the following criteria: eigenvalues indicating the percentage of explained variance, scree plot, and the rotated component matrix. Upon thorough examination of the performed analysis steps, a two-factor solution emerged as the best fit, accounting for 47.08% of the total variance. The overall KMO value was 0.72, signifying that the sample size used for factor analysis was appropriate. Bartlett’s test of sphericity was significant, with χ^2^(66) = 321.127, *p* < 0.001. Seven items were excluded either due to moderate loadings (< 0.5) on two factors (items 1, 8, 11, and 12) or because they didn’t load on any factor (items 2, 9, and 17). Both factors consisted of six items each and demonstrated satisfactory internal consistency. Specifically, Cronbach’s alpha coefficient for the first factor (eigenvalue = 3.67), was 0.78, while the second factor (eigenvalue = 1.98), had a coefficient of 0.73. The first factor was named “Simple Strategies” because the items associated with it describe the utilization of external aids or the implementation of simple cognitive regulatory processes, such as organizing information. The second factor was named “Complex Strategies” because the related items describe more intricate and demanding cognitive strategies that necessitate greater effort and involve complex information processing, such as mental imagery and story creation. MMQ-Strategies subscale results can be found in Table 2.

**TABLE 2:** Exploratory factor analysis for the Multifactorial Memory Questionnaire-Strategies subscale.

Items	Simple Strategies	Complex Strategies
10. How often do you make a list, such as a grocery list or a list of things to do?	0.80	
15. How often do you write down in a notebook things that you want to remember?	0.80	
5. How often do you write things on a calendar, such as appointments or things you need to do?	0.77	
18. How often do you write a note or reminder for your-self (other than on a calendar or in a notebook)	0.60	
7. How often do you organize information you want to re- member; for example, organize your grocery list according to food groups?	0.56	
19. How often do you mentally retrace your steps to remember something, such as the location of a misplaced item?	0.53	
4. How often do you create a visual image of some- thing you want to remember, like a name and a face?		0.69
14. How often do you create a story to link together information you want to remember?		0.68
3. How often do you create a rhyme out of what you want to remember?		0.65
16. How often do you create an acronym out of the first letters in a list of things to remember, such as carrots, apples, and bread (cab)?		0.64
6. How often do you go through the alphabet one letter at a time to see if it sparks a memory for a name or word?		0.63
13. How often do you repeat something to yourself at increasingly longer and longer intervals so that you will remember it?		0.57
Eigenvalue	3.67	1.98
% of variance	30.61%	16.47%
Reliability (Cronbach’s α)	0.78	0.73

### 2.5 Statistical analysis

Statistical analysis was performed using SPSS Statistics, Version 27 (IBM Corp. Released 2020. IBM SPSS Statistics for Macintosh, Version 27.0. Armonk, NY: IBM Corp). A series of one-way analysis of variance (ANOVA) were conducted to test group differences in metacognitive beliefs and strategy use. To estimate effect size partial eta-squared (η^2^) was used. A p-value < 0.05 indicated statistical significance.

## 3 Results

### 3.1 Group differences in MKEM, MKEA, MKEEFs, and MMQ-Strategies

First, mean scores for each factor-underlying dimension of the provided scales were calculated and then a series of ANOVAs were performed to assess if there were any differences between the two groups in terms of their offline metacognitive scores. The dependent variables represented the following mean scores: MKEM, MKEA-Divided and Shifted Attention, MKEA-Concentration, MKEEFs-Planning, MKEEFs-Inhibition, MMQ-Complex Strategies, and MMQ-Simple Strategies; group was identified as the independent variable with two levels (aMCI, HC).

The results revealed significant differences between the two groups. Specifically, there were significant differences for the MKEM scale, F(1,97) = 16,09, p < 0.001, η2 = 0.142, and for MKEA-Divided and Shifted Attention dimension of the MKEA, F(1,97) = 16,91, p < 0.001, η2 = 0.148. These findings suggest that individuals with aMCI perceive themselves as performing less effectively than HC in everyday situations related to memory and to divided and shifting attention. No statistically significant differences were observed for the remaining variables (see Table 3).

**TABLE 3:** Group differences in metacognitive beliefs of efficacy and use of cognitive strategies.

	aMCI (*n* = 50)	HC (*n* = 50)			
	**Mean (SD)**	**Mean (SD)**	**F**	**p**	**η ^2^**
MKEM^1^	3.14(0.39)	3.43(0.32)	16.088	< 0.001	0.142
**MKEA^2^**					
Divided and Shifted attention	3.37(0.33)	3.62(0.28)	16.911	< 0.001	0.148
Concentration	2.68(0.53)	2.77(0.51)	0.635	n.s.	0.007
**MKEEFs^3^**					
Inhibition	3.54(0.38)	3.60(0.34)	0.717	n.s.	0.007
Planning	3.68(0.33)	3.73(0.28)	0.921	n.s.	0.009
**MMQ-Strategies^4^**					
Simple	2.29(0.87)	2.16(0.88)	0.547	n.s.	0.006
Complex	0.92(0.64)	0.94(0.74)	0.008	n.s.	0.000

^1^MKEM, Metacognitive Knowledge for Everyday Memory.

^2^MKEA, Metacognitive Knowledge for Everyday Attention.

^3^MKEEFs, Metacognitive Knowledge for Everyday Executive Functions.

^4^MMQ-Strategies, Multifactorial Memory Questionnaire, subscale Strategies.

## 4 Discussion

The present study aimed to explore the metacognitive beliefs of people with aMCI regarding their efficacy in various everyday life situations, serving as an index of their metacognitive awareness about their cognitive abilities. Additionally, the study aimed to assess the frequency of applying cognitive strategies to compensate for their cognitive deficits.

Concerning the first hypothesis of the present study, statistically significant differences between aMCI and HC were only observed for the metamemory scale and the MKEA’s subscale “Divided/Shifted Attention”. This could be attributed to the pronounced and identifiable memory deficits that characterize the aMCI group, as memory complaints form one of the diagnostic criteria (Petersen, 2004).

Tasks requiring divided or shifted attention are tasks with high attentional load demands, typically challenging for older adults. These tasks are challenging for older adults due to cognitive changes inherent with aging, which include reductions in cognitive flexibility, working memory, and attentional control, all necessary for such tasks (Bopp and Verhaeghen, 2018). Age-related slowing in information processing speed (Salthouse, 2019), diminished synaptic plasticity (Morrison and Baxter, 2012) and alterations in brain connectivity (Fjell et al., 2016; Ramanoël et al., 2018) further compound these difficulties, making it more challenging for older adults to quickly switch between tasks or manage multiple tasks at the same time. As a result, individuals with aMCI are likely to experience heightened difficulties in these situations compared to those in the HC group, leading to their deficits becoming more easily noticeable. However, besides the detected differences the aMCI group still perceived their performance in the related scenarios as good enough.

Interestingly, although no significant differences were observed between the two groups for the concentration subscale of the MKEA questionnaire, both groups reported lower efficacy ratings compared to the other scales. Both groups perceive their ability to sustain attention on a specific task or stimulus over time as moderate. This observation aligns with existing studies on cognitive aging, according to which older adults tend to get distracted more easily making it for them more difficult to remain focused for a prolonged period of time (Weeks and Hasher, 2014). These difficulties were equally perceived by the two groups. These findings prompt consideration of two important factors: inhibitory control and frontal aging. Age-related decline in inhibitory control may result in increased vulnerability to distracting information entering working memory, offering a possible explanation for the attentional challenges experienced by both groups (Tan et al., 2019; Bessette et al., 2020). Additionally, the prefrontal cortex, crucial for attentional control, undergoes age-related changes known as frontal aging (Petrican et al., 2017; Chong et al., 2019), including reductions in gray matter volume (Manard et al., 2016; Ramanoël et al., 2018) and alterations in connectivity (Fjell et al., 2016; Chong et al., 2019; Perinelli et al., 2022). Hence, such changes could impact individuals’ focusing abilities, irrespective of their cognitive health status.

However, as regards metacognitive beliefs of efficacy in everyday tasks involving inhibitory control and planning skills, no significant differences were observed between the aMCI and HC groups and both groups reported good to very good performance. The absence of significant differences, alongside the high efficacy scores from the aMCI group could receive several explanations. First it could reflect their relatively mild symptoms and the real-world situations described in the questionnaires may not be sufficiently challenging for them. Moreover, in contrast to the isolated cognitive demands of experimental tasks, real-life situations often require the simultaneous engagement of multiple cognitive skills. This allows aMCI participants to perform well by compensating with their still intact abilities. Furthermore, individuals with aMCI may not have the specific metacognitive knowledge needed to understand the more complex aspects of cognitive functioning, such as executive functions. This could result in struggles when trying to identify difficulties within these cognitive domains. This particular metacognitive-knowledge-gap might not be unique to aMCI individuals but could also be common in the general population. As a result, people with aMCI often face criticism or feedback from their environment related to their forgetfulness, while feedback about a decline in their abilities to think flexibly or to control their behavior may be rarely given or entirely overlooked, even though these deficits exist as evidenced by neuropsychological studies assessing executive functions in individuals with aMCI (Ávila et al., 2015; Chehrehnegar et al., 2020; Rattanavichit et al., 2022) and received further support from neuroimaging studies showing functional abnormalities within frontoparietal brain areas that construct the neural basis of cognitive control and regulation processes (Li et al., 2015; Sheng et al., 2017; Zhao et al., 2022). Another possible explanation about these findings could be that the scenarios described in the questionnaire mirror typical and relatively simple daily activities, implying that routine execution might eliminate the apparent impact of cognitive challenges. Also, these scenarios are relatively simple, aligning with the level of cognitive impairment in our aMCI group, which has not yet advanced to severe cognitive deterioration. Finally, the questionnaire’s phrasing could also influence self-perception. By asking about the ability to perform tasks rather than their difficulty level, the aMCI participants may still perceive themselves as capable despite facing increased challenges, leading to the reported high scores.

The findings regarding the use of cognitive strategies are in line with our hypothesis. Specifically, the results showed no significant differences in strategy use between the two groups, which aligns with previous research (Farias et al., 2018). Despite reporting lower efficacy in memory and divided/shifted attention tasks, individuals with aMCI do not report to utilize cognitive strategies more frequently than their cognitively healthy counterparts, underscoring the struggle of those with aMCI to effectively manage their difficulties. However, as previously mentioned, despite reporting lower self-efficacy compared to HC, individuals with aMCI perceive their performance in related everyday life scenarios as quite competent. Therefore, they may not feel the necessity to utilize more or different cognitive strategies than usual. Moreover, both groups exhibited a preference for simple strategies over complex mental strategies, suggesting an inclination toward methods that are easier to implement and less mentally demanding. However, this tendency could also suggest a lack of understanding or awareness about more effective but complex cognitive strategies, especially among individuals with aMCI. Previous studies have shown the benefits of cognitive training programs in enhancing metacognitive knowledge and strategies for older adults and those with MCI (Bailey et al., 2010; Hertzog et al., 2012; Vrani et al., 2013; Moro et al., 2015; Youn et al., 2020). Specifically, metacognitive training programs that concentrate on memory knowledge and the application of cognitive strategies can offer significant advantages to older adults. They grant the understanding of how and when to employ these cognitive strategies in the most effective manner. The importance of boosting self-efficacy in older adults should not be underestimated, as this can inspire them to learn more challenging, yet more effective cognitive strategies, while also improving their overall well-being (McDougall et al., 2010; Wiegand et al., 2013). Therefore, it could be more beneficial and efficient for a training program to begin by enhancing self-efficacy and then proceed to target control and regulatory skills (Bampa et al., 2021). Without this approach, negative beliefs about one’s cognitive abilities may jeopardize cognitive engagement and the motivation to learn and incorporate new cognitive strategies.

Considering the study’s limitations, future research could integrate feedback from caregivers or relatives and use objective performance evaluations regarding different cognitive domains to better understand metacognitive awareness in MCI population. Also, the study sample was composed of highly educated individuals, which could imply enhanced metacognitive abilities, thus a deeper insight into their cognitive difficulties. The symptoms exhibited by the aMCI participants in our study were relatively mild when viewed across the severity spectrum of MCI symptoms. Thus, these findings might not be universally applicable, particularly in relation to populations with varying educational backgrounds or those with more severe cognitive impairment.

## 5 Conclusion

The present study revealed specific differences between aMCI and HC in terms of their beliefs about their ability to perform daily life situations, particularly those requiring memory and divided or shifted attention. However, there seems to be a gap in understanding or applying effective cognitive strategies for compensation. This emphasizes the potential value of cognitive training programs focusing on enhancing metacognitive knowledge and the practical use of cognitive strategies. Such interventions could greatly assist individuals with MCI in improving their cognitive management skills, thereby enhancing their autonomy, self-efficacy, and overall quality of life. Interestingly, individuals with aMCI overall reported high self-efficacy scores which warrants careful consideration. Future research should aim to set a “gold standard” for metacognitive awareness, determining when high self-efficacy beliefs are beneficial, serving as a positive reinforcement for cognitive engagement and effort, and when it indicates a negative aspect signifying a deficiency of insight and potential engagement in risky behaviors. Moreover, having accurate self-efficacy alone might not be the optimal solution. Pure awareness without the acquisition of appropriate tools, such as training in effective strategies or other regulatory behaviors, might only lead to increased distress and may not be beneficial for individuals with MCI. Therefore, a comprehensive approach that integrates both metacognitive awareness and metacognitive regulation could potentially provide the most beneficial support for these individuals.

## Data availability statement

The raw data supporting the conclusions of this article will be made available by the authors, without undue reservation.

## Ethics statement

The studies involving humans were approved by the Scientific Committee of “Alzheimer Hellas.” The studies were conducted in accordance with the local legislation and institutional requirements. The participants provided their written informed consent to participate in this study.

## Author contributions

GB: Conceptualization, Data curation, Methodology, Project administration, Writing–original draft, Writing–review and editing. DM: Supervision, Writing–review and editing. PM: Writing–review and editing. EM: Methodology, Writing–review and editing. GP: Resources, Writing–review and editing. MS: Resources, Writing–review and editing. GK: Resources, Writing–review and editing. EP: Resources, Writing–review and editing. MT: Resources, Validation, Writing–review and editing.

## Footnotes

1
https://www.baycrest.org/Baycrest_Centre/media/content/MMQ-Greek.pdf
