# Supplementary material for: Metacognitive beliefs of efficacy about daily life situations and use of cognitive strategies in amnestic mild cognitive impairment: a cross-sectional study
Source: Front Psychol. 2024 Feb 13;15:1275678. doi: 10.3389/fpsyg.2024.1275678 (PMC10896964; doi:10.3389/fpsyg.2024.1275678)
Supplement: Supplementary file 1 [file Data_Sheet_1.docx]

Supplementary Material

| **Supplemental Table 1.** *Exploratory Factor Analysis for the Metacognitive Knowledge for Everyday Memory (MKEM) scale* | | | | |  |  |
| --- | --- | --- | --- | --- | --- | --- |
| Items | Everyday Memory | | | |  |  |
| Imagine that you want to call someone. You read the phone number to memorize it. How well do you manage to remember the phone number without looking at it again? | | | .62 | |  |  |
| Imagine that someone (i.e., a friend, son/daughter etc.) asks you what you did yesterday. How well do you manage to remember what you did the previous day? | | | .68 | |  |  |
| Imagine that you are watching TV and in fifteen minutes you need to remember to turn off the boiler. In order to do so you set a reminder, for instance your alarm. How well do you manage to remember what it is you need to do when you hear the alarm? | | | .54 | |  |  |
| Imagine that you are on your way, and you ask a passer-by for directions in order to find the address you are looking for. How well do you manage to remember the directions you received until you find your destination? | | | .60 | |  |  |
| Imagine that are calling to arrange a doctor’s appointment for the next week. How well do you manage to remember the appointment (by memory)? | | | .74 | |  |  |
| Imagine that you want to tell a story that you read earlier in a book or in a newspaper. How well do you manage to remember details of that story such as names, place, time? | | | .63 | |  |  |
| Imagine that at the end of the week you need to pay a bill. How well do you manage to remember it (by memory)? | | | .64 | |  |  |
| Imagine that you ask for an address. You are looking for a pen to write it down. How well do you manage to remember the address until you find the pen? | | | .71 | |  |  |
| Imagine that you are asking a member of your family how he/she is going to spend the day. How well do you manage to remember what he/ she told you without asking again? | | | .67 | |  |  |
| Imagine that you are watching TV. A member of your family has asked you to call him/ her when the news start. How well do you manage to remember to call him/ her when the news start? | | | .67 | |  |  |
| Imagine that you are at the supermarket’s counter check and the cashier tells you the amount you need to pay. In the meantime, you are looking for your wallet. How well do you manage to remember the amount you need to pay until you open your wallet? | | | .61 | |  |  |
| How well do you manage to remember details (i.e., names, place, time) from a conversation you had earlier? | | | .73 | |  |  |
| Eigenvalue | | | | 5.15 | | |
| % of variance | | | | 42.95 | | |
| Reliability | | | | .88 | | |

Bampa G, Kouroglou D, Metallidou P, Tsolaki M, Kougioumtzis G, Papantoniou G, et al. Metacognitive Scales: Assessing Metacognitive Knowledge in Older Adults Using Everyday Life Scenarios. Diagnostics. 2022 Oct;12(10):2410.

| **Supplemental Table 2.** *Exploratory factor analysis for the Metacognitive Knowledge for Everyday Attention (MKEA) scale* | | |
| --- | --- | --- |
| Items | Divided & Shifted Attention | Concentration |
| How well do you manage to follow a conversation in which more than two people participate? | .71 |  |
| Imagine that you arrive at the train or bus station a lot earlier from your departure time. As a result, you are walking around the station to spend your spare time. How well do you manage to not lose your route announcement? | .69 |  |
| Image that you are at the bank, and you are waiting for your number to appear on the announcement table. How well do you manage to stay focused so that you don’t lose your turn when your number appears? | .69 |  |
| How well do you manage to have a conversation while you are cooking or driving? | .59 |  |
| Imagine that you are having a conversation, suddenly another person enters the room and asks you something irrelevant, for example the time. How well do you manage to answer the question and then continue the conversation you had from the point you left it? | .55 |  |
| Imagine that you are waiting for the streetlights to turn green while at the same time you are talking on your phone. How well do you manage to initiate crossing the street when the lights turn green? | .53 |  |
| Imagine that you are making your groceries list and suddenly the phone rings. You stop to answer the phone. After turning off the phone, how well do you manage to continue your list from the point you left it? | .51 |  |
| How well do you manage to concentrate on a task you need to complete without getting distracted by a problem you are dealing with (work or personal life related)? |  | .76 |
| How well do you manage to concentrate on reading a text (i.e., book, magazine, newspaper), when there is noise (i.e., noise from the street or kids playing)? |  | .70 |
| Image that you are reading a large text (at least 2 pages long). How well do you manage to remain concentrate until the end? |  | .68 |
| How well do you manage to watch a TV program and talk with someone at the same time? |  | .65 |
| How well do you manage to read something while listening to music? |  | .61 |
| Eigenvalue | 3.99 | 1.45 |
| % of variance | 33.26 % | 12.08 % |
| Reliability | .74 | .75 |

Bampa G, Kouroglou D, Metallidou P, Tsolaki M, Kougioumtzis G, Papantoniou G, et al. Metacognitive Scales: Assessing Metacognitive Knowledge in Older Adults Using Everyday Life Scenarios. Diagnostics. 2022 Oct;12(10):2410.

| **Supplemental Table 3.** *Exploratory Factor Analysis for the Metacognitive Knowledge for Everyday Executive Functions (MKEEFs) scale* | | |
| --- | --- | --- |
| Items | Planning | Inhibition |
| Imagine that you have planned to go on a walk with a friend, but it starts raining. How well do you manage to think of an alternative plan considering the weather (i.e., sit on a cafeteria)? | .80 |  |
| Imagine that you are on the phone and suddenly someone is knocking at your door. How well do you manage to turn off the phone and open the door? | .75 |  |
| Imagine that you are having a conversation with someone and suddenly the phone rings. How well do you manage to pause the conversation and answer the phone? | .66 |  |
| Imagine that you want to attend a theater performance or to go on a trip with the bus. How well do you manage to call and book a ticket for the time and date that works better for you? | .65 |  |
| Imagine that you have planned with friends or family to go out for dinner. You enter at a restaurant but there are no free tables. How well do you manage to think of an alternative place? | .41 |  |
| Imagine that it is the beginning of the month, and you just got your salary/pension. How well do you manage to handle the money to cover the month’s expenses? |  | .75 |
| Imagine that you are at a room where you need to keep quiet (i.e., at a doctor’s office or at a library). How well do you manage to not talk loudly? |  | .60 |
| Imagine that you are at a dinner gathering with family and/or friends. How well do you manage to stop yourself from drinking or eating something that your doctor has forbidden you? |  | .63 |
| Imagine that you are at the bank, and someone steals your turn. How well do you manage to not react in a rude way? |  | .61 |
| Imagine that you decide to go on a trip this weekend. How well do you manage to make your suitcase with the staff you are going to need? |  | .44 |
| Eigenvalue | 3.50 | 1.50 |
| % of variance | 31.81 % | 13.64% |
| Reliability | .70 | .65 |

Bampa G, Kouroglou D, Metallidou P, Tsolaki M, Kougioumtzis G, Papantoniou G, et al. Metacognitive Scales: Assessing Metacognitive Knowledge in Older Adults Using Everyday Life Scenarios. Diagnostics. 2022 Oct;12(10):2410.

**
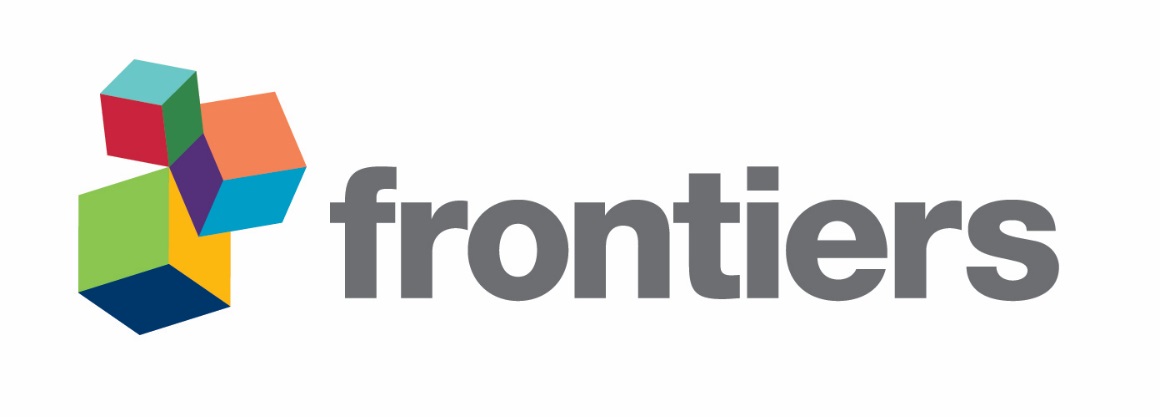
**
